# Effect of oral nitrates on pulse pressure and arterial elasticity in patients aged over 65 years with refractory isolated systolic hypertension: study protocol for a randomized controlled trial

**DOI:** 10.1186/1745-6215-14-388

**Published:** 2013-11-14

**Authors:** Daniel Abad-Pérez, Blanca Novella-Arribas, Francisco J Rodríguez-Salvanés, Luis M Sánchez-Gómez, Iluminada García-Polo, Carmen Verge-González, Carmen Suárez-Fernández

**Affiliations:** 1 Servicio de Medicina Interna, Hospital Universitario de la Princesa, Diego de León 62, Planta 10, 28006, Madrid, Spain; 2 D.G. de Atención Primaria, SERMAS Consejería de Sanidad, Comunidad de Madrid, Instituto de Investigación Sanitaria del Hospital Universitario de La Princesa (IP) Red Temática de Investigación en Enfermedades Cardiovasculares (RECAVA), Pza. Carlos Trías Bertrán 7, Madrid 28020, Spain; 3 Unidad de Información Clínico Asistencial, Servicio de Admisión y Documentación Clínica, Hospital Universitario de la Princesa Instituto de Investigación sanitaria del Hospital Universitario de La Princesa (IP) Red Temática de Investigación en Enfermedades Cardiovasculares (RECAVA), Diego de León 62, Madrid 28006, Spain; 4 Agencia de Evaluación de Tecnología Sanitarias (AETS), ISCIII Instituto de Investigación Sanitaria del Hospital Universitario de La Princesa (IP) Red de Investigación en Servicios de Salud en Enfermedades Crónicas (REDISSEC), Monforte de Lemos 5, Madrid 28029, Spain

**Keywords:** Hypertension, Nitrates, Older people, Systolic-hypertension

## Abstract

**Background:**

Isolated systolic hypertension is a highly prevalent disease among the elderly. The little available evidence on the efficacy of nitrates for treating the disease is based on small experimental studies.

**Methods/design:**

We performed a multicenter, randomized, double-blind, phase III, placebo-controlled trial in 154 patients aged over 65 years with refractory isolated systolic hypertension. Patients were randomized to placebo or 40 mg/day of extended-release isosorbide mononitrate added to standard therapy and titrated to 60 mg/day at week 6 if blood pressure exceeded 140/90 mmHg.

The primary objective was to assess the effect on clinical pulse pressure of extended-release isosorbide mononitrate added to standard therapy in patients aged over 65 years with refractory isolated systolic hypertension after 3 months of treatment.

The secondary objectives were as follows: to quantify the effect of adding the study drug on central blood pressure and vascular compliance using the augmentation index and pulse wave velocity; to evaluate the safety profile by recording adverse effects (frequency, type, severity) and the percentage of patients who had to withdraw from the trial because of adverse events; to quantify the percentage of patients who reach a clinical systolic blood pressure <140 mmHg or <130 mmHg measured by ambulatory blood pressure monitoring; and to quantify the change in pulse pressure measured by ambulatory blood pressure monitoring.

**Discussion:**

Few clinical trials have been carried out to test the effect of oral nitrates on isolated systolic hypertension, even though these agents seem to be effective. Treatment with extended-release isosorbide mononitrate could improve control of systolic blood pressure without severe side effects, thus helping to reduce the morbidity and mortality of the disease.

**Trial registration:**

EUDRACT Number: 2012-002988-10

## Background

Hypertension is responsible for many deaths worldwide [1,2]. It is very prevalent in Spain and is estimated to affect more than 25% of adults [3,4]. In the United States, more than 60% of people aged over 65 years are hypertensive. The most prevalent type of hypertension is isolated systolic hypertension (ISH), which has a frequency that increases with age and can reach over 80% in octogenarian patients [5]. Loss of arterial elasticity accompanied by increased arterial stiffness is the main pathogenic mechanism in ISH, which is characterized by high systolic blood pressure (SBP) (≥140 mmHg) and normal diastolic blood pressure (DBP) (<90 mmHg), with a consequent increase in differential pressure, or pulse pressure (PP), the main predictor of cardiovascular complications in older people [6]. The mechanism responsible for the decrease in arterial compliance is the subject of debate and has been considered to be purely structural (that is, secondary to the calcification produced by age in the walls of large arteries) or partly functional (that is, due to endothelial dysfunction and increased smooth muscle tone).

Treating ISH is beneficial, as it can reduce both cardiovascular morbidity and total mortality [7], even in much older people [8]. Besides the widespread recommendation to reduce SBP to less than 140 mmHg, there is no evidence from clinical trials to support this recommendation in older people, since none of the studies demonstrating a benefit included patients with an SBP between 140 and 160 mmHg [9]. No first-line antihypertensive treatments can produce a clinically relevant reduction in arterial compliance and, therefore, PP, [10,11], with the result that treatment options are limited. The decrease in SBP is accompanied by declines in DBP that can compromise coronary perfusion and increase the risk of coronary complications, which is again a limiting factor for current treatments. It seems reasonable to assume that a drug that decreases both SBP and PP could have additional benefits in the treatment of ISH. The value of such an approach could be proven in the short term by demonstration of changes in vascular function parameters (augmentation index (AI) and pulse wave velocity (PWV)) and in the long term by documenting a reduction in cardiovascular morbidity and mortality.

Nitrates have been used for many years to treat patients with angina pectoris, acute coronary syndrome, and acute lung edema. These agents are not generally used to treat hypertension, except in emergency cases, in which case they are used intravenously to produce a rapid decrease in blood pressure, although tachyphylaxis occurs within a few days of treatment. All nitrates, as donors of nitric oxide (NO), produce intense arterial vasodilation associated with direct effects on smooth muscle cells, by activating guanylyl cyclase to form cGMP, which inhibits calcium entry into the cell. The most common adverse events are headache and hypotension, although dizziness, nausea, fatigue, weakness, and tachycardia can also be observed.

Currently available tools enable us to evaluate vascular function and central hemodynamics non-invasively. Central blood pressure (CBP) can be measured in larger arteries using pulse wave analysis, and available data suggest a closer relationship between CBP and cardiovascular morbidity and mortality [12]. Additionally, PWV, which is the best parameter for measuring arterial elasticity [13,14], can be measured non-invasively and provides an accessible and exhaustive evaluation of arterial stiffness, which is the underlying pathogenic mechanism of ISH [15].

Our literature review revealed only five mini-trials assessing the effect of nitrates on blood pressure (BP) and control of ISH. Sample sizes were small and the studies were not performed in the context of refractory ISH [16-20]. The results provide some evidence of the possible use of exogenous NO donors to decrease the reflected wave amplitude and PP [16-22]. Use of nitrates could have a beneficial effect on the control of ISH (as SBP falls with almost no change in DBP), with a possible reduction in cardiovascular morbidity and mortality.

Therefore, we designed a study to evaluate the effect on PP and vascular function (CBP, AI, and PWV) of the addition of extended-release isosorbide mononitrate to standard treatment in older patients with refractory ISH. Refractory hypertension is defined as uncontrolled hypertension in patients using three or more antihypertensive drugs (one of which is a diuretic) at the maximum dosage; we changed our protocol to include patients receiving low doses of diuretic and maintaining at least two more drugs, since most patients never use diuretics at the maximum dosage (for example, hydrochlorothiazide at 50 mg/day).

If a beneficial effect is demonstrated, a study of morbidity and mortality would be justified.

### Hypothesis

The addition of extended-release isosorbide mononitrate to standard antihypertensive treatment in patients with refractory ISH produces a positive effect on vascular function, resulting in reduced PP and improved CBP, AI, and PWV.

### Aims

The primary objective of our study was to compare the effect of extended-release isosorbide mononitrate on clinical PP with that of a placebo, in addition to the standard treatment, in patients over 65 with refractory ISH after 3 months of treatment.

The secondary goals were as follows:

1. To quantify the effect of extended-release isosorbide mononitrate on vascular function (estimated using CBP, AI, and PWV).

2. To evaluate the safety profile by estimating the frequency, type, and severity of adverse events and the percentage of patients who had to withdraw from the study because of adverse events, in particular headache and orthostatic hypotension.

3. To determine the percentage of patients who reach clinical SBP <140 mmHg in both treatments groups.

4. To determine the percentage of patients who reach a 24-hour mean SBP <130 mmHg (measured using ambulatory blood pressure monitoring (ABPM)) in both treatment groups.

5. To measure the change in PP using ABPM.

## Methods/design

Ours is a phase III, multicenter, randomized, double-blind, placebo-controlled trial performed in several primary care centers in the Regional Community of Madrid and in Hospital La Princesa, Madrid. The trial is registered in the EU Clinical Trials Register (EUDRACT Number: 2012-002988-10), and the protocol was approved by the Clinical Research Ethics Committee of the Regional Community of Madrid.

The study was designed to evaluate the efficacy and safety of a marketed drug in a therapeutic indication different from that for which it is approved. The objective of the study was to evaluate, after 3 months of appropriate treatment, the effect of adding extended-release isosorbide mononitrate to standard treatment in patients with resistant ISH. No recommendations have been made on the treatment of these patients; therefore, we designed a study to compare the net effect of the study drug with that of placebo.

### The study began in January 2013 and is ongoing

#### Participants

The study population comprises patients aged ≥65 years of both genders attended at primary care centers or at the Hypertension Unit of Hospital La Princesa. All patients give their written informed consent to participate in the study. Patients are included if they meet all the inclusion criteria and none of the exclusion criteria. Table 1 shows the inclusion and exclusion criteria .

**Table 1:** Inclusion and exclusion criteria

**Inclusion criteria**	**Exclusion criteria**
Aged 65 or over	Current treatment with nitrates, intolerance to them or contraindication to their use
Refractory ISH: SBP ≥140 mmHg and DBP <90 mmHg and SBP in ABPM ≥130 mmHg, despite treatment with three drugs at maximum dosage, one of them diuretic, during at least one month before the start	Concomitant treatment with phosphodiesterase 5 inhibitors
	SBP ≥180 mmHg
Good adherence to treatment, defined as a good response to all questions of Morisky-Green’s test	Permanent atrial fibrillation
	Secondary hypertension
Life expectancy greater than 1 year	Congestive heart failure, grade III-IV NYHA
Signing the informed consent	Chronic kidney disease with glomerular filtration rate ≤30 ml/min, estimated by MDRD
Ability to understand study procedures and to comply with them for the entire length of the study	
	Liver insufficiency
	Active cancer
	Anemia ≤8 g/dl
	Simultaneous participation in another clinical trial
	Not signing the informed consent
	Any other circumstance that, according to the research criteria, advises against the inclusion to the study

Patients are included if they meet all the inclusion criteria and none of the exclusion criteria.

ABPM: ambulatory blood pressure monitoring, DBP: diastolic blood pressure, ISH: isolated systolic hypertension, NYHA: New York heart association, MDRD: modification of diet in renal disease, SBP: systolic blood pressure.

#### Variables

Variables reported for trial participants include the following:

1. Demographic characteristics: sex, age, height, weight, body mass index, waist circumference, and smoking and drinking habits, at screening.

2. Antihypertensive treatment and concomitant treatments, at screening.

3. Medical history: presence of macrovascular or microvascular disease and diabetes mellitus or dyslipidemia.

4. Laboratory tests: hematology and biochemistry with kidney and liver function markers to assess that the patient meets all the inclusion criteria (screening visit).

5. BP measurement at baseline, 6 weeks, and completion (SBP, DBP, PP, and heart rate (mean of 3) while seated and 1 minute after standing). Monitoring is performed between 8 am and 10 am, before patients take their medication.

6. 24-hour ABPM (Spacelabs device, OSI systems company, US; http://www.spacelabshealthcare.com/): mean of SBP, DBP, heart rate, and PP during activity and at rest (baseline and completion). Patients take their antihypertensive medication as usual.

7. CBP, PWV, and AI at visits 1 and 3 (measured with the patient lying supine using a Sphygmocor device, AtCor Medical, Australia; http://www.atcormedical.com/).

8. Adherence to treatment (Morisky-Green test) at screening and at visits two and four.

9. Side effects: any adverse event during administration of the study drug, in particular headache and orthostatic hypotension.

#### Randomization, blinding, and assignment to treatment groups

Subject numbers are assigned sequentially as each patient enters the study. Subjects are assigned to the study drug through a randomization schedule based on the randomization plan. The study drug is stored under the conditions specified on the label in a locked, safe area of the pharmacy department to prevent unauthorized access.

Both the study drug and the placebo are indistinguishable; they are manufactured by the same company and are similar in appearance, organoleptic characteristics, and presentation.

In the event of an emergency, the investigator decides whether it is necessary to unblind the subject’s treatment assignment using the unblinding envelopes provided to the hospital and/or pharmacy. If unblinding is necessary, the investigator or study pharmacist must record the reason for unblinding, as well as the date and time of the event.

During the study, the events considered sufficient reason for unblinding are as follows: admission to hospital or visit to the emergency department related to the study drug. The patients can be removed from the study without unblinding in the following circumstances: withdrawal of consent, major adverse event(s), ineligibility (arising either during the study or retrospectively after having been overlooked at screening), significant protocol deviation, disease progression requiring discontinuation of the study medication or inability to continue to comply with study procedures, loss to follow-up, or death. Each participant has the right to withdraw from the study at any time, for any reason, and with no repercussions for his/her medical care.

In the case of unblinding, the analyses are based on an intent-to-treat population, which includes all randomized subjects. Because the percentage of lost participants was predicted in the calculation of sample size, lost patients need not be replaced during follow-up. The person analyzing the results remains blind to the patient’s group.

#### Trial intervention

When all of the procedures at the inclusion visit are performed, the patients are sent to the investigator responsible for assessing vascular function (PWV, AI, and CBP), randomization, and delivery of the study medication (12-week supply). Vascular function parameters are measured by the same trained investigator to ensure consistency and homogeneity.

Before administration of the drug, the investigator explains the most common adverse events (headache and orthostatic hypotension) to the patient.

After randomization, a single 40-mg dose of extended-release isosorbide mononitrate is administered between 9:30 and 10:30 am during the first 6 weeks. Study patients attend three visits after randomization. The first is at 6 weeks from baseline, when clinical parameters are assessed. These include BP (seated and standing), resting pulse, and the Morisky-Green test. All adverse events are recorded, regardless of their relationship to the study medication. If the objective (SBP <140 mmHg) is not reached, the dose is titrated to 60 mg/d (single dose); otherwise, the 40-mg dose is maintained.

After 12 weeks of treatment, the study is considered to have finished, except in the case of patients who withdraw prematurely. At the final visit, the parameters recorded are PP, SBP, DBP, heart rate and ambulatory BP. Compliance is also evaluated using the Morisky-Green test, and the investigator questions the patient about adverse events, orthostatic hypotension, and other treatments taken during the study. Vascular function parameters (CBP, AI, and PWV) are measured between 2 and 5 days before the final visit while the patient is still taking the medication.

**Figure 1:**
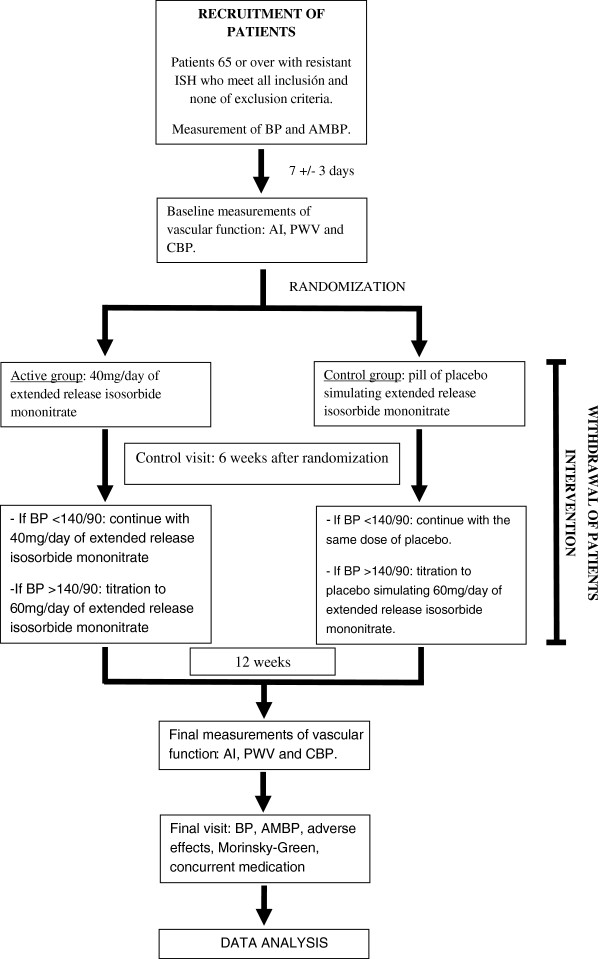
Study flow-chart.

Figure 1 shows the study flow-chart.

#### Evaluation of effectiveness

The study drug is considered effective if PP decreases significantly with respect to placebo according to the primary objective.

Secondary efficacy is assessed based on the improvement in vascular function parameters, the percentage of patients achieving control of BP at the clinic and with ABPM, and the change in PP measured by ABPM. All objectives are measured based on the comparison of values from the baseline and final visits.

#### Ethics

The planning and conduct of this trial are subject to national legislation. The trial is performed in accordance with the Guidelines of the World Medical Association (WMA), the Declaration of Helsinki (1996), the Guidelines of GCP (CPMP/ICH/135/95), and the stipulations of national drug and data protection laws and other applicable regulatory requirements.

The investigator is responsible for ensuring that no patient undergoes any study-related examination or activity before either the patient or the legal guardian has signed an informed consent document. Detailed information is provided to the patient/legal guardian before consent is given.

The investigator must inform the patient of the aims, methods, anticipated benefits, and potential hazards of the study, including any discomfort it may entail. The investigator must inform patients that in providing informed consent, they are giving permission for representatives of the trial site coordinating investigator or regulatory authorities to inspect their medical records to verify the information collected. The patient must be given every opportunity to clarify any points he/she does not understand. Patients or legal guardians must sign and date the informed consent form. Patients who refuse to give or withdraw their written informed consent are either not included or do not continue to participate in the study.

In accordance with Spanish legislation (RD 223/2004 of 6 February), a civil liability insurance policy has been taken out to cover possible adverse events arising from the study medication that could affect the trial subjects.

#### Evaluation of safety

The most common adverse events are headache, orthostatic hypotension, somnolence, dizziness, and asthenia. These generally disappear with continued treatment. Less common or rare adverse events include bradycardia, angina pectoris, acute hypotension and syncope, nausea, vomiting, exfoliative dermatitis, allergic reactions, and flushing. Overdose can produce hypotension, tachycardia, cyanosis, shock, seizures, vomiting, agitation, respiratory failure, bradycardia, and methemoglobinemia.

The period for reporting an adverse event starts when the study drug is administered and ends with the last follow-up visit. The association between an adverse event and the trial medication must be classed as related or not related to the study drug by a study physician.

Adverse events considered related to the study medication are monitored until resolution or until the event is considered stable. All related adverse events that result in a participant’s withdrawal from the study or are present at the end of the study should be monitored until resolution. It is left to the investigator’s clinical judgment whether or not an adverse event is of sufficient severity to warrant the participant’s withdrawal from treatment.

This study is performed without an external sponsor under the coordination of the scientific committee and under the supervision of a safety committee, which is blind to the intervention. The safety committee is composed of three physicians, each of whom is an expert in hypertension. The committee also includes a biostatistician and expert in bioethics.

#### Sample size calculation

Sample size is predetermined based on the effect the variable PP, which is the primary objective. According to the results of the Starmans-Kool trial [17], the PP after treatment could be 87 mmHg in the placebo group and 79 mmHg in the experimental group, both with a standard deviation of 16 mmHg. In order to obtain a power of 80% to detect differences using a *t* test for independent samples, with an α risk of 5%, the sample size for each group must be 64 patients. Considering losses of 20% of recruited individuals, which could weaken the sample for some secondary objectives, we decided to increase the sample size by this percentage, although all the tests are performed by intent to treat. For this reason, the final sample size for each group is 77 patients.

#### Statistical analysis

The statistical analysis will comprise the following parts:

1. In descriptive analysis of the sample, quantitative variables will be expressed as measures of central tendency (mean or median) and dispersion (standard deviation or interquartile range) for symmetrical distributions; normality will be tested using Kolmogorov-Smirnov test. Appropriate transformations will be applied in cases of a non-normal distribution. Qualitative variables will be expressed as proportions with their standard error.

2. Baseline characteristics, especially those that could influence the results, will be compared using the *t* test or Mann–Whitney test for quantitative variables and the Pearson chi-squared or Fisher exact test for qualitative variables. The main variables that could influence the result of this study at initiation are age, PP, SBP, and DBP. In the case of inequality, these factors will be treated as confounding variables in the final efficacy analysis.

3. The differences between the means and their confidence interval (CI), or the median ratio and their CI, will be used as the main measure of the effect. The relative risk, absolute risk reduction, and relative risk reduction will be used as a measure of the effect for the qualitative variables. A 95% CI will be calculated for all estimations.

4. To control for possible confounding variables, linear models or linear regression will be applied for dependent quantitative variables and logistic regression models for dependent qualitative variables.

5. In all the analyses, the null hypothesis tested will be the lack of effect (difference of means = 0, RR = 1) between the experimental and the control groups. Missing data will be processed using a sensitivity analysis.

6. As this is a two-arm, placebo-controlled, parallel study, no subgroups will be analyzed to measure secondary endpoints and no interim analysis will be performed.

## Discussion

ISH remains difficult to control in many cases, despite the availability of various types of appropriate medication; however, most are able to reduce SBP, albeit with a considerable associated reduction in DBP. The consequent severe side effects, such as reduced coronary perfusion and orthostatic hypotension, limit treatment options.

Hypertension continues to be the most important cardiovascular risk factor today, and cardiovascular illnesses are the main cause of death in developed countries, especially among people over 65.

The results of our trial should demonstrate whether treatment with isosorbide mononitrate improves not only control of arterial pressure, but also vascular function in patients with refractory ISH. If our hypotheses are proved, a clinical trial with morbidity and mortality as the main end-points would be justified. Such a trial could also be used to investigate the long-term effects of this drug.

Few clinical trials have been carried out to test oral nitrates in patients with ISH, even though these agents seem to be effective and have been used over long periods in the treatment of other illnesses, such as ischemic heart disease, hypertensive emergency, or acute lung edema.

Our trial is performed on a collaborative basis between the hospital (center for recruitment and vascular function tests) and the primary care center, because most of these patients have their main contact with the health service through their primary care physicians. A collaboration of this type makes it easier to reach patients and provides more robust results with greater external validity.

Our study is subject to three limitations. First, potentially high loss to follow-up because of non-fatal side effects; however, this is compensated for, as it was taken into account in the sample size calculation. Second, in the long term, patients could develop tolerance to nitrates, although this was not demonstrated in any of the trials we reviewed. Finally, our trial was not designed to demonstrate reductions in morbidity and mortality; rather, our main objective was to evaluate tolerance and effects on some intermediate variables.

In conclusion, treatment of ISH with extended-release isosorbide mononitrate could improve control of SBP with minimal side effects, thus helping to reduce the morbidity and mortality of the disease.

## Trial status

The trial is in the recruitment phase.

## Abbreviations

AE: Adverse event; AI: Augmentation index; AR: Adverse reaction; BP: Blood pressure; CBP: Central blood pressure; CI: Confidence interval; CRF: Case report form; DBP: Diastolic blood pressure; ISH: Isolated systolic hypertension; NO: Nitric oxide; PP: Pulse pressure; PWV: Pulse wave velocity; SBP: Systolic blood pressure.

## Competing interests

All the authors declare that they have no competing interests.

## Authors’ contributions

DAP participated in the design and coordination of the study, performed the vascular function measurements, and drafted the manuscript. BNA participated in the design and coordination of the study and helped to draft the manuscript. FJRS participated in the design of the study, performed the sample size calculations, and designed the statistical analysis. IGP participated in the design and coordination of the study and helped to draft the manuscript. As Principal Investigator, she also recruited patients. LMSG participated in the design and coordination of the study, performed the sample size calculations, and designed the statistical analysis. CVG participated in the design and coordination of the study. CSF conceived the study, participated in its design and coordination, and helped to draft the protocol and manuscript. She also recruited patients. All the authors read and approved the final version of the manuscript. The NISH group is composed of: Daniel Abad Pérez, María Jesús Fernández Luque, Ángela Gallego Arenas, Iluminada García Polo, Gema Gil Juberias, Amelia González Gamarra, Pilar Loeches Belinchón, Javier López González, Soledad Mayayo Vicente, Blanca Novella Arribas, Francisco José Rodríguez Salvanés, Lourdes Ruíz Díaz, Félix Mata Benjumea, Milagros González Béjar, Francisco López Corral, Marta Ruíz López, Rosa Sánchez Alcalde, Luis María Sánchez Gómez, Belén Sierra García, Carmen Suárez Fernández and Carmen Verge González. (DAP, MJFL, AGA, IGP, GGJ, AGG, PLB, JLG, SMV, BNA, FJRS, LRD, FMB, MGB, FLC, MRL, RSA, LMSG, BSG, CSF and CVG).

## Authors’ information

EUDRACT Number: 2012-002988-10.

## References

[B1] Lopez AD Mathers CD Ezzati M Jamison DT Murray CJ Global and regional burden of disease and risk factors, 2001: systematic analysis of population health data Lancet 2006 367 1747 1757 10.1016/S0140-6736(06)68770-9 16731270

[B2] Lewington S Clarke R Qizilbash N Peto R Collins R Prospective studies collaboration Age-specific relevance of usual blood pressure to vascular mortality: a meta-analysis of individual data for one million adults in 61 prospective studies Lancet 2002 360 1903 1913 12493255 10.1016/s0140-6736(02)11911-8

[B3] Banegas JR Rodríguez-Artalejo F de la Cruz Troca JJ Guallar-Castillón P del Rey Calero J Blood pressure in Spain: distribution, awareness, control, and benefits of a reduction in average pressure Hypertension 1998 32 998 1002 10.1161/01.HYP.32.6.998 9856963

[B4] Banegas JR Rodríguez-Artalejo F Ruilope LM Graciani A Luque M de la Cruz-Troca JJ García-Robles R Tamargo J Rey-Calero J Hypertension magnitude and management in the elderly population of Spain J Hypertens 2002 20 2157 2164 10.1097/00004872-200211000-00014 12409953

[B5] Franklin SS Milagros JJ Wong ND L’Italien GJ Lapuerta P Predominance of isolated systolic hypertension among middle-aged and elderly US hypertensives: analysis based on national health and nutrition examination survey (NHANES) III Hypertension 2001 37 869 874 10.1161/01.HYP.37.3.869 11244010

[B6] Gasowski J Fagard RH Staessen JA Grodzicki T Pocock S Boutitie F Gueyffier F Boissel JP INDANA project collaborators: pulsatile blood pressure component as predictor of mortality in hypertension: a meta-analysis of clinical trial control groups J Hypertens 2002 20 145 151 10.1097/00004872-200201000-00021 11791038

[B7] Staessen JA Gasowski J Wang JG Thijs L Den Hond E Boissel JP Coope J Ekbom T Gueyffier F Liu L Kerlikowske K Pocock S Fagard RH Risks of untreated and treated isolated systolic hypertension in the elderly: meta-analysis of outcome trials Lancet 2000 355 865 872 10.1016/S0140-6736(99)07330-4 10752701

[B8] Beckett NS Peters R Fletcher AE Staessen JA Liu L Dumitrascu D Stoyanovsky V Antikainen RL Nikitin Y Anderson C Belhani A Forette F Rajkumar C Thijs L Banya W Bulpitt CJ HYVET Study Group Treatment of hypertension in patients 80 years of age or older N Engl J Med 2008 358 1887 1898 10.1056/NEJMoa0801369 18378519

[B9] Mancia G Laurent S Agabiti-Rosei E Ambrosioni E Burnier M Caulfield MJ Cifkova R Clément D Coca A Dominiczak A Erdine S Fagard R Farsang C Grassi G Haller H Heagerty A Kjeldsen SE Kiowski W Mallion JM Manolis A Narkiewicz K Nilsson P Olsen MH Rahn KH Redon J Rodicio J Ruilope L Schmieder RE Struijker-Boudier HA van Zwieten PA European society of hypertension: reappraisal of European guidelines on hypertension management: a European society of hypertension task force document J Hypertension 2009 27 2121 2158 10.1097/HJH.0b013e328333146d 19838131

[B10] Schiffrin EL Deng Li Y Comparison of effects of angiotensin I-converting enzyme inhibition and beta-blockade for 2 years on function of small arteries from hypertensive patients Hypertension 1995 25 699 703 10.1161/01.HYP.25.4.699 7721419

[B11] Ghiadoni L Magagna A Versari D Kardasz I Huang Y Taddei S Salvetti A Different effect of antihypertensive drugs on conduit artery endothelial function Hypertension 2003 41 1281 1286 10.1161/01.HYP.0000070956.57418.22 12719441

[B12] Agabiti-Rosei E Mancia G O’Rourke MF Roman MJ Safar ME Smulyan H Wang JG Wilkinson IB Williams B Vlachopoulos C Central blood pressure measurements and antihypertensive therapy: a consensus document Hypertension 2007 50 154 160 10.1161/HYPERTENSIONAHA.107.090068 17562972

[B13] Willum-Hansen T Staessenn JA Torp-Pedersen C Rasmussen S Thijs L Ibsen H Jeppesen J Prognostic value of aortic pulse wave velocity as index of arterial stiffness in the general population Circulation 2008 113 664 670 16461839 10.1161/CIRCULATIONAHA.105.579342

[B14] Laurent S Boutouyrie P Asmar R Gautier I Laloux B Guize L Ducimetiere P Benetos A Aortic stiffness is an independent predictor of all-cause and cardiovascular mortality in hypertensive patients Hypertension 2001 37 1236 1241 10.1161/01.HYP.37.5.1236 11358934

[B15] Laurent S Cockcroft J Van Bortel L Boutouyrie P Giannattasio C Hayoz D Pannier B Vlachopoulos C Wilkinson I Struijker-Boudier H European network for non-invasive investigation of large arteries. Expert consensus document on arterial stiffness: methodological issues and clinical applications Eur Heart J 2006 27 2588 2605 10.1093/eurheartj/ehl254 17000623

[B16] Pauca AL Kon ND O’Rourke MF Benefit of glyceryl trinitrate on arterial stiffness is directly due to effects on peripheral arteries Heart 2005 91 1428 1432 10.1136/hrt.2004.057356 15761047 PMC1769166

[B17] Starmans-Kool MJ Kleinjans HA Lustermans FA Kragten JA Breed JG Van Bortel LM Treatment of elderly patients with isolated systolic hypertension with isosorbide in an asymmetric dosing schedule J Hum Hypertens 1998 12 557 561 10.1038/sj.jhh.1000664 9759991

[B18] Duchier J Iannascoli F Safar M Antihipertensive effect of sustained-release isosorbide dinitrate for isolated systolic systemic hypertension in the elderly Am J Cardiol 1987 60 99 102 3300248 10.1016/0002-9149(87)90993-3

[B19] Stokes GS Ryan M Brnabic A Nyberg G A controlled study of the effects of isosorbide mononitrate on arterial blood pressure and pulse wave form in systolic hypertension J Hypertens 1999 17 1767 1773 10.1097/00004872-199917120-00015 10658944

[B20] Felizardo A Maldonado J Pego M Teixeira F Providência L Os nitratos na modulaçao farmacológica das ondas reflectidas e sua importância no tratamento da hipertensâo arterial do idoso Rev Port Cardiol 1997 16 607 611 9432207

[B21] Stokes GS Nitrates as adjunct antihypertensive treatment Curr Hypertens Rep 2006 8 60 68 10.1007/s11906-006-0042-7 16600161

[B22] Stokes GS Barin ES Gilfillan KL Effects of isosorbide mononitrate and angiotensin II inhibition on pulse wave reflection in hypertension Hypertension 2003 41 297 301 10.1161/01.HYP.0000049622.07021.4F 12574098

